# Duodenal Ganglioneuroma: A Rare Tumor Causing Upper Gastrointestinal Bleed

**DOI:** 10.1055/s-0041-1735644

**Published:** 2021-09-14

**Authors:** Arkadeep Dhali, Sukanta Ray, Gopal Krishna Dhali, Ranajoy Ghosh, Avik Sarkar

**Affiliations:** 1 Department of GI Surgery, School of Digestive and Liver Diseases, Institute of Postgraduate Medical Education and Research, Kolkata, West Bengal, India; 2 Department of Gastroenterology, School of Digestive and Liver Diseases, Institute of Postgraduate Medical Education and Research, Kolkata, West Bengal, India; 3 Department of GI Pathology, School of Digestive and Liver Diseases, Institute of Postgraduate Medical Education and Research, Kolkata, West Bengal, India; 4 Department of GI Radiology, School of Digestive and Liver Diseases, Institute of Postgraduate Medical Education and Research, Kolkata, West Bengal, India

**Keywords:** duodenum, ganglioneuroma, periampullary globular mass, upper gastrointestinal hemorrhage

## Abstract

Neuroblastic tumors (NTs) include neuroblastoma, ganglioneuroblastoma, and ganglioneuroma (GN). They are very rare in adults. The Surveillance, Epidemiology, and End Results identified 144 patients ≥20 years old at diagnosis (6.1%) from 1973 to 2002. GNs account for 14% of all localized NT. Since 1957, a total of four cases of GN of the duodenum have been reported. We report a novel case of GN of the periampullary region in the duodenum in a 41-year-old man presenting with chronic upper gastrointestinal bleed. Given the rarity of GNs in this age group and the nonspecificity of radiological features, this diagnosis is often missed until histopathology is done. This may negatively affect the prognosis of an otherwise well-prognosticated disease.

A 41-year-old man presented with complaints of passage of tarry black loose stool for the last 6 months. It was intermittent, well tolerated and with no postural hypotensive symptoms. It was associated with dull abdominal pain over the epigastrium which occurred before meals and relieved after taking food. There was no prior history of blood transfusion. There was no history of hematemesis, jaundice, abdominal pain, abdominal distension, hematochezia, fever, loss of weight, loss of appetite, altered bowel, and bladder habits. No history of previous gastrointestinal (GI) bleeds, peptic ulcer, and blood dyscrasia. No history of usage of anticoagulants, iron supplementation, or analgesics. Physical examination revealed pallor with no other localizing signs. Provisional diagnosis at this stage was peptic ulcer disease, variceal bleed, or malignancy.

Laboratory evaluation showed hemoglobin of 8.1 g/dL. Other blood counts, liver function tests, renal function tests, and hematological parameters were within normal limits. Stool occult blood was detected. Ultrasonography showed a mildly prominent hepatomegaly with an otherwise normal study. Upper GI endoscopy and colonoscopy were normal. Side view endoscopy showed a large globular periampullary mass with smooth and erythematous overlying mucosa and bleeding point. There were no ulcer seen at the lesion site ( Fig. 1 ). Computed tomography (CT) scan ( Fig. 2 ) showed a polyp arising from second part of duodenum, ∼4 cm × 2 cm with no significant adenopathy. Main pancreatic duct was not dilated. Mild hepatomegaly with fatty liver was seen. Rest of the study was normal. Differential diagnoses included gastrointestinal stromal tumor (GIST), duodenal adenocarcinoma, lymphoma, and carcinoid tumor. CA 19-9 level was 437 U/mL. Endoscopic ultrasound guided biopsy was attempted; however, it was abandoned due to excessive bleeding during procedure. Patient was later stabilized.

**Fig. 1:**
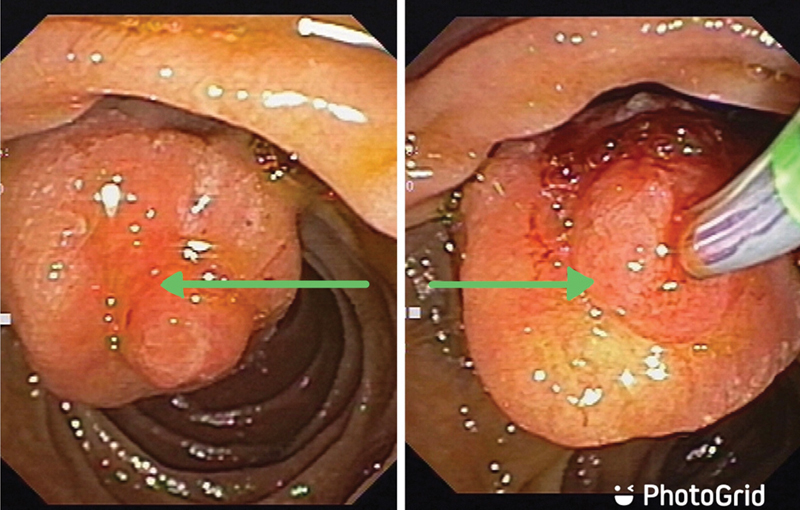
Side view endoscopy showing (green arrow) a large periampullary globular mass with smooth and erythematous overlying mucosa and bleeding point.

**Fig. 2:**
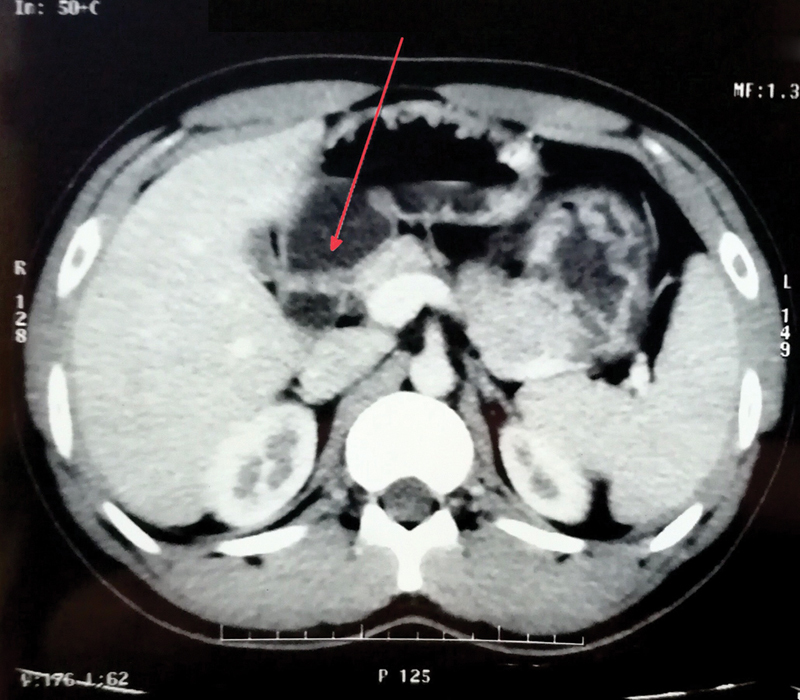
Computed tomography scan showing (red arrow) a polyp arising from second part of duodenum, ∼4 cm × 2 cm with no significant adenopathy.

Due to the suspicion of malignant growth and need for biopsy, surgical options were explained to the patient. After informed consent, patient was taken up for duodenotomy, D1–D2 junction polypectomy, feeding jejunostomy, and cholecystectomy. Surgery was done under general anesthesia. We found a mass ∼2 cm, with a wide-based pedicle, arising from the medial wall of the D1–D2 junction ( Fig. 3 ). Drain amylase levels was 88 IU/L. Resected specimen was sent for histopathological examination and showed mature ganglion cells interspersed in a fibrillary background suggestive of ganglioneuroma (GN) ( Figs. 4 and 5 ). Immunohistochemistry showed that the tumor was positive for S-100 ( Fig. 6 ) and chromogranin A which confirmed the diagnosis. Postoperative period was uneventful. Patient was discharged on postoperative day 5. Monthly follow-up was done for the next 2 years and there was no evidence of recurrence of symptoms.

**Fig. 3:**
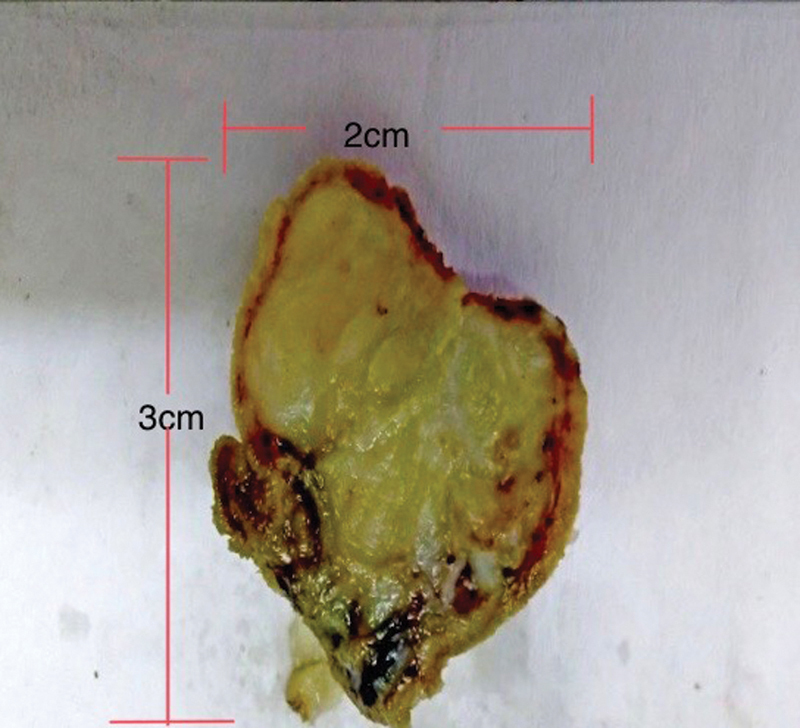
Gross: globular well-circumscribed mass with myxoid stroma.

**Fig. 4:**
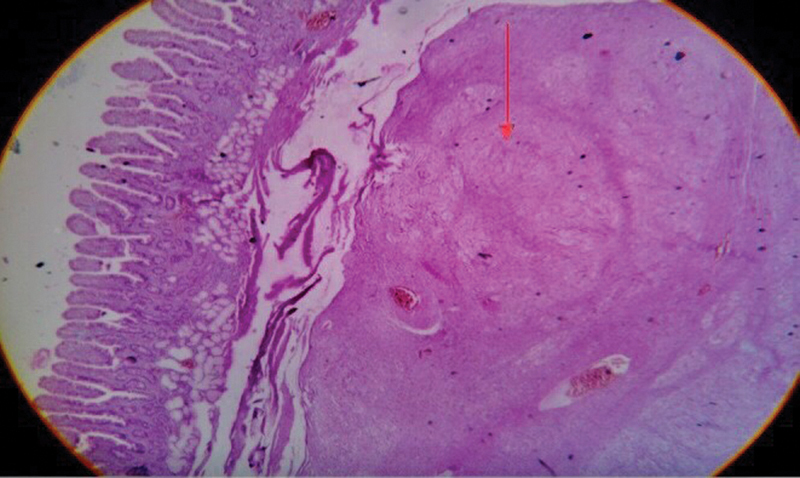
Hematoxylin and eosin stain (×40): circumscribed vague nodular mass in submucosa with fibrillary appearance (red arrow).

**Fig. 5:**
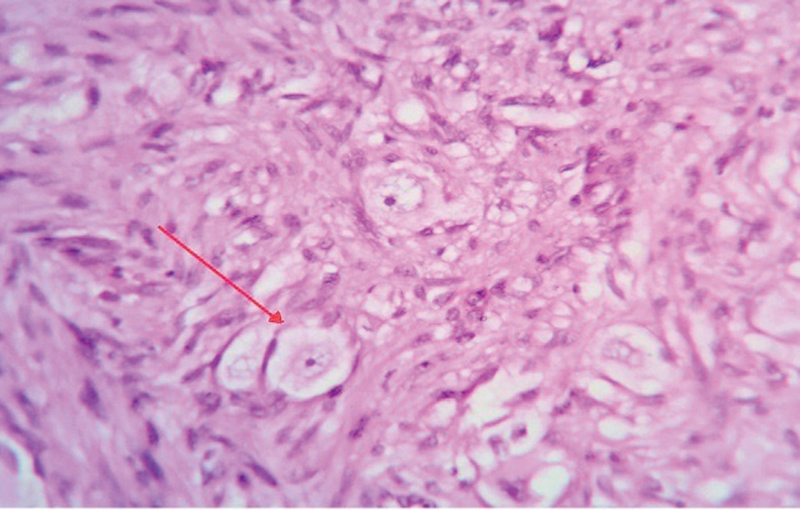
Hematoxylin and eosin stain (×400): scattered ganglion cells in a fibrillary background (red arrow).

**Fig. 6:**
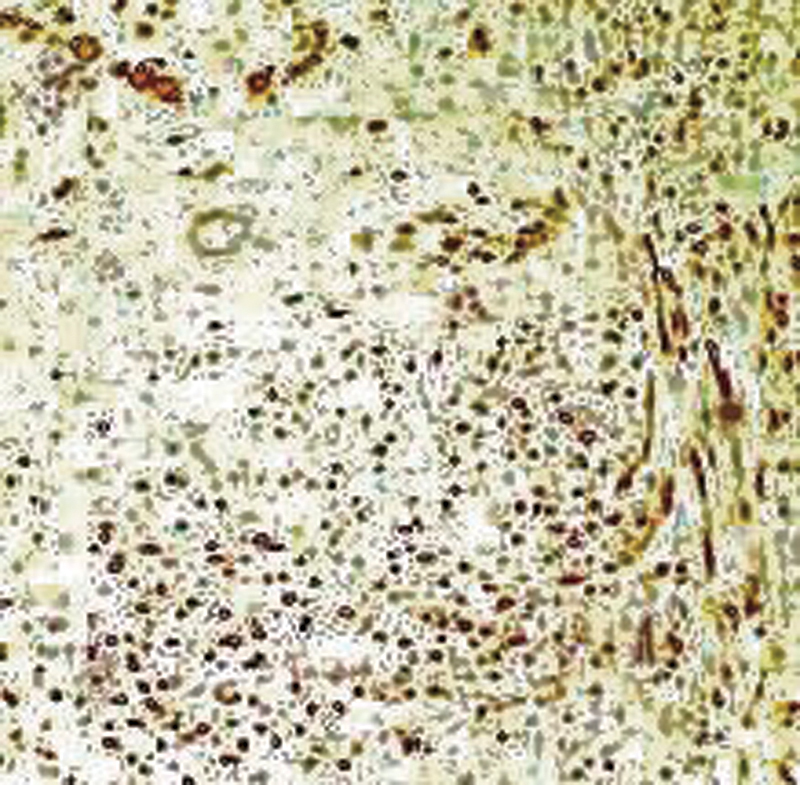
Immunohistochemical staining is positive for S-100 protein.

## Discussion

Neuroblastic tumors (NTs) include neuroblastoma, ganglioneuroblastoma (nodular or intermixed [GNBN/GNBI]), and GN. These tumors originate from neural crest cells and range from immature, undifferentiated tumors to mature differentiated ones. According to the International Neuroblastoma Pathology Classification (INPC), GNBI and GN represent the mature end of a spectrum. 1 NTs are the most common extracranial solid tumors in childhood. 2 The median age of diagnosis of NT is 17 months and >90% of patients are diagnosed before 5 years of age. 3 4 Most frequent symptoms include pain, palpable tumor mass, and reduced general condition. Patients with GN tend to present less frequently with adrenal tumors and show positive metaiodobenzylguanidine (MIBG) uptake and elevated urine catecholamine metabolites less frequently when compared with immature NT (NB/GNBN). Outcome is excellent for GN when tumor is adequately resected. Additional chemotherapy is not warranted. 5 Incomplete resection with minor residuals (<2 cm) is also shown to have no effect on prognosis indicating that resection does not have to be radical for the treatment of GN. Instead, subtotal resection without endangering vital structures seems to be sufficient. 6 7 8

NTs are very rare in adults. The Surveillance, Epidemiology, and End Results identified 144 patients ≥20 years old at diagnosis (6.1%) between 1973 and 2002 and 35 patients ≥60 years old at diagnosis (0.9%) between 1973 and 2010. 9 10 Adolescents and adults with NB are reported to have a worse outcome than children. 9 11

GN is a well-differentiated benign tumor composed of mature ganglion cells and neurofibers. About 60% of GNs occur in <20 years of age. 12 13 14 GNs account for 14% of all localized NT. 5 A total of 74 paragangliomas have been reported till date. This includes GN, nonchromaffin paraganglioma, gangliocytic paraganglioma, paraganglioma, and paraganglioneuroma. GNs of the duodenum are a very rare entity with only four cases having been reported till now. 12 13 14 15 In the duodenum, they are most commonly located at the second or third part with a predilection for the ampulla of Vater. They most commonly present with features of upper GI bleeding due to erosion of the top portion of the mass. However, due to their rarity, they are often missed to more common differentials such as variceal bleed or peptic ulcer disease. This may negatively affect the prognosis of an otherwise well-prognosticated disease leading to poor outcomes.

Radiological investigations are often inconclusive. Although larger and malignant lesion with lymph node involvement can be picked up in cross-sectional imaging such as CT scan, endoscopy may reveal a polypoid mass but in some instances like the above case, due to its small size, lesion may be missed on an upper GI endoscopy and require a side viewing endoscopy. Biopsy via endoscopy may be attempted leading to incomplete resection of the tumor with major residuals (>2 cm). Moreover, biopsy findings at this stage may be misleading as the tumor is submucosal in origin and endoscopic biopsy mostly takes mucosal sample. 16 Due to the benign nature of the lesion, treatment may be stopped at this stage. However, while duodenal paragangliomas have been originally thought to be only benign in nature, few cases have shown recurrence or lymph node metastasis with the epithelioid cells showing malignant potential. 17 18 This may adversely affect the outcome. Other associated syndromes such as von Recklinghausen's disease and neurofibromatosis must also be kept in mind.

Treatment options include either local excision or pancreaticoduodenectomy. The latter is reserved for larger tumors where excision is not possible. Radiotherapy has no role in management.

In histopathology, careful observation is required for ganglion cells and neurofibrillary background, as they are very important to rule out other close differentials of polypoidal lesions of the duodenum such as GIST, Brunner's gland hamartoma, adenocarcinoma, lymphoid, carcinoid, neuroendocrine tumor, and other NTs. 19 Gangliocytic paragangliomas have epithelioid cells in addition to ganglion cells and neurofibers. The other differentials lack ganglion cells. All these differentials respond differently; hence, a definitive pathological diagnosis is required to treat and prognosticate the disease appropriately. Moreover, histopathological features such as nuclear atypia, increased mitotic activity, and infiltrative tumor margins suggest aggressive behavior of GN. 20

## Conclusion

Herein, we have reported the case to highlight the obstacles in coming to a diagnosis. Clinical presentation is often unclear and radiological investigations may be nonspecific leading to a diagnostic dilemma. I-MIBG scan or urinary catecholamines is not necessary, and hence, their value as a diagnostic determiner have diminished. What was novel about this case other than its extremely rare occurrence was the age of presentation, as GNs are usually found in <20 years of age. Therefore, in the adult population, such as the case presented here, an open mind is required to come to the diagnosis. Additionally, there is also a consensus to highlight the need for optimal surgical resection to ensure best prognosis. For surveillance assessment, we performed monthly follow-up with microscopic examination for stool occult blood and annual endoscopy which were normal. Hence, adequate surgical resection ensures less likelihood of recurrence in this tumor.
